# Crystal structure of a new polymorph of (2*S*,3*S*)-2-amino-3-methyl­penta­noic acid

**DOI:** 10.1107/S2056989018006126

**Published:** 2018-05-01

**Authors:** Sofia Curland, Elena Meirzadeh, Yael Diskin-Posner

**Affiliations:** a Department of Materials and Interfaces, Weizmann Institute of Science, Israel; b Chemical Research Support Unit, Weizmann Institute of Science, Israel

**Keywords:** crystal structure, isoleucine, polymorph, amino acid

## Abstract

A new polymorph of (2*S*,3*S*)-2-amino-3-methyl­penta­noic acid, l-isoleucine C_6_H_13_NO_2_, crystallizes in the monoclinic space group *P*2_1_ with four independent mol­ecules in the asymmetric unit. The mol­ecules are *zwitterions*. In the crystal, N—H⋯O hydrogen bonds link two pairs of independent mol­ecules and their symmetry-related counterparts to form two types of layers stacked in an *anti-parallel* manner parallel to (001). The hydro­phobic aliphatic isopropyl groups protrude from these layers.

## Abstract 2

A new polymorph of (2*S*,3S)-2-amino-3-methyl­penta­noic acid, l-isoleucine C_6_H_13_NO_2_, crystallizes in the monoclinic space group *P*2_1_ with four independent mol­ecules in the asymmetric unit. In the crystal, N–H⋯O hydrogen bonds link two pairs of independent mol­ecules and their symmetry-related counterparts to form two types of layers stacked in an *anti-parallel* manner parallel to (001). The hydro­phobic aliphatic isopropyl groups protrude from these layers.

## Chemical context   

(2*S*,3*S*)-2-Amino-3-methyl­penta­noic acid, known as l-isoleucine (l-Ile), is one of the 20 amino acids common in animal proteins and required for normal functioning in humans. l-Ile is classified as a hydro­phobic amino acid and is one of the two common amino acids that has a chiral side chain. l-Ile is essential for human muscle tissue recovery after exercise, along with Valine and Leucine.
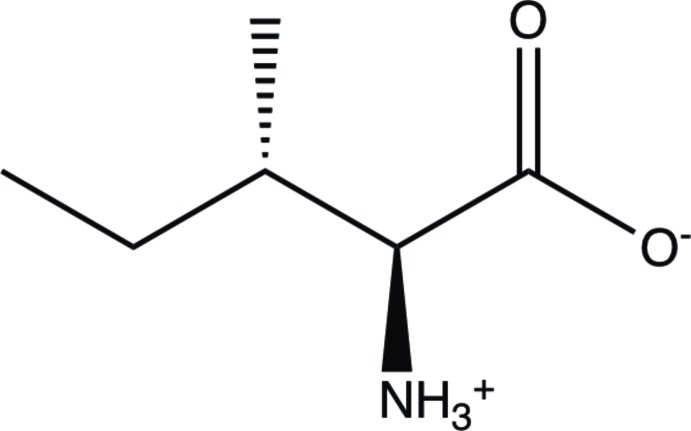


The structure of l-Ile was first determined by Torii & Iitaka (1971 ▸). The crystal was found to belong to the monoclinic space group *P*2_1_, with four mol­ecules in the unit cell, *Z* = 4. The asymmetric unit contains two independent mol­ecules, with the side chain of the l-Ile mol­ecules exhibiting two different conformations (Görbitz & Dalhus, 1996 ▸; Torii & Iitaka, 1971 ▸). Another polymorph in the ortho­rhom­bic space group *P222_1_* with the unit cell containing eight mol­ecules was reported by Khawas (1970 ▸). The presence of an additional l-Ile polymorph is supported by X-ray powder diffraction measurements by Anuar *et al.* (2009 ▸), who suggested that l-Ile is prone to polymorphism as a result of the structural thermal motion of the aliphatic side chain.

## Structural commentary   

**Figure 1:**
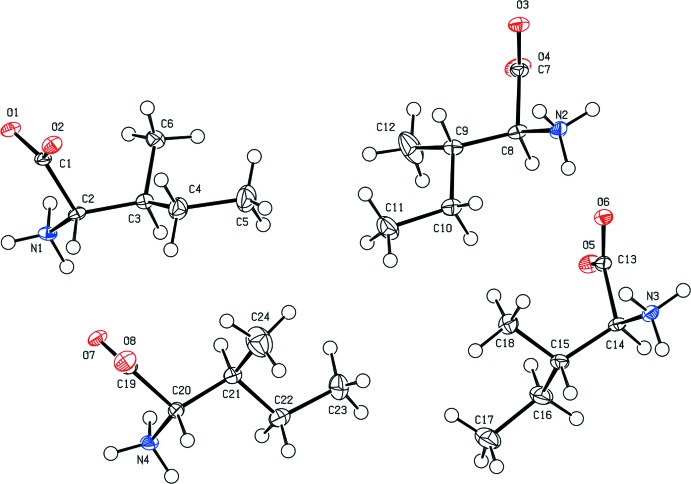
The asymmetric unit of the title compound with atomic numbering. Displacement ellipsoids are shown at the 50% probability level.

**Figure 2:**
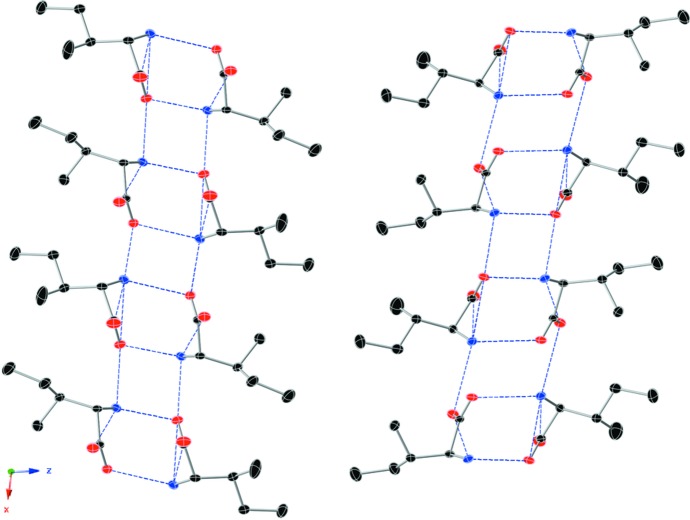
Part of the crystal structure viewed perpendicular to the *ac* plane showing adjacent *anti-parallel* layers formed by hydrogen-bonded pairs and symmetry-related mol­ecules. The hydro­phobic side chains protrude away and stack together. Displacement ellipsoids are shown at the 50% probability level (C atoms black, O red, N blue). H atoms are omitted for clarity. Blue dashed lines denote hydrogen bonds.

In the structure of the title compound there are four l-Ile mol­ecules in the asymmetric unit (Fig. 1 ▸). The mol­ecules are zwitterions and organized in pairs. The hydro­philic parts of the mol­ecules are facing each other and generate inter­molecular N—H⋯O hydrogen bonds (Table 1 ▸), within the pair and with symmetry-related pairs. The aliphatic parts of the mol­ecules are exposed, pointing away from the hydrogen-bonded network, creating a hydro­phobic layer (Fig. 2 ▸). A similar network pattern was described previously (Görbitz & Dalhus, 1996 ▸; Torii & Iitaka, 1971 ▸).

The existence of another chiral center in the side chain allows for conformational differences. Each l-Ile pair consists of two types of conformers. This is presented in the values of the following torsion angles. The two mol­ecules of conformer type I have torsion angles N1—C2—C3—C6 = 80.1 (4)°, N1—C2—C3—C4 = −155.4 (3)° and N3—C14—C15—C18 = 78.1 (4)°, N3—C14—C15—C16 = −155.8 (3)°. The other two mol­ecules are of conformer type II with the torsion angles N2—C8—C9—C12 = 178.6 (4)°, N2—C8—C9—C10 = −56.9 (5)° and N4—C20—C21—C24 = 179.1 (4)°, N4—C20—C21—C22 = −56.8 (5)°. Furthermore, there is a minor conformational variance between all the four independent mol­ecules, as illus­trated by the torsion angles of the *iso-*propyl side chains: C6—C3—C4—C5 = −56.6 (5)°, C12—C9—C10—C11 = −51.6 (6)°, C18—C15—C16—C17 = −58.9 (5)° and C24—C21—C22—C23 = −53.2 (6)°.

## Supra­molecular features   

In the crystal, N—H⋯O hydrogen bonds (Table 1 ▸) connect the mol­ecules, forming layers parallel to (001). The polar side of l-Ile is embedded inside the layers while the side chains are oriented away, creating a hydro­phobic surface. However, this hydrogen-bonding network has directionality along the polar *b* axis and specifically parallel to (001) (see Figs. 2 ▸ and 3 ▸). The adjacent layer is slightly rotated and grows in the opposite direction to the first one, an *anti-parallel* layer. The structure is composed of alternating layers with the hydro­philic side generating a hydrogen-bonding network growing in the opposite direction and the hydro­phobic side chains are directed outside. There is a slight offset between the layers to allow the hydro­phobic side chains to fit the gaps in the adjacent layer surface.

## Database survey   

**Figure 4:**
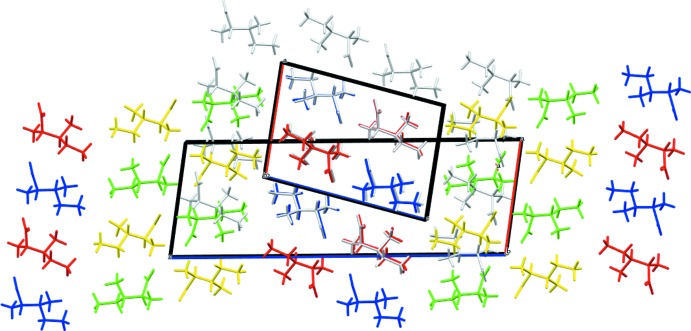
Overlay of two structures with mol­ecules presented as capped sticks along the *b* axis. The previous monoclinic *P2_1_* polymorph with two mol­ecules in the asymmetric unit is the small unit cell with all mol­ecules colored in gray and ordered in a *parallel* layer arrangement. The new monoclinic *P2*_1_ polymorph has four mol­ecules in the asymmetric unit (colored red, blue, yellow and green). The colors are according to symmetry equivalence. While the blue and red pairs form exactly the same network layer as the polymorph reported by Görbitz & Dalhus (1996 ▸), it is evident that the green and yellow pairs have a different orientation, with an *anti-parallel* layer arrangement.

A comparison between the polymorph presented in this paper and the one reported by Görbitz & Dalhus (1996 ▸) is presented in Fig. 4 ▸. Both structures have the same monoclinic crystallographic *P*2_1_ symmetry; however, one has four mol­ecules in the unit cell and the other has only two. As described above, the layers show growth directionality and a pair of l-Ile mol­ecules manage the layer organization. The new polymorph has alternating layers in opposite direction, *anti-parallel*, unlike the polymorph reported by Görbitz & Dalhus (1996 ▸), that has only *parallel* layers.

## Synthesis and crystallization   

Single crystals of l-Ile were grown from supersaturated aqueous solutions, *via* slow evaporation at 323 K in a clean-room environment. The l-Ile powder (Holand–Moran 99%) was dissolved in water (Ultra-pure Millipore water, 18.2 MΩ cm at 298 K, Millipore Synergy UV, Type 1 water) by heating to 353 K, with constant stirring until total dissolution. The hot solution was then filtered through cotton wool into glass crystallization dishes, which were covered with filter paper in order to allow slow evaporation, placed in a heating bath. Colorless crystal chunks, suitable for X-ray crystallographic analysis were obtained. The absolute configuration of the title compound is already known.

## Refinement   

Crystal data, data collection and structure refinement details are summarized in Table 2 ▸. H atoms were placed in calculated positions with C—H = 0.98–1.00 Å, N—H = 0.91 Å and included in the refinement in a riding-model approximation with *U*_iso_(H) = 1.2*U*_eq_(C) or 1.5*U*_eq_(N, C_meth­yl_).

## Supplementary Material

Crystal structure: contains datablock(s) I. DOI: 10.1107/S2056989018006126/lh5872sup1.cif

Structure factors: contains datablock(s) I. DOI: 10.1107/S2056989018006126/lh5872Isup2.hkl

Supporting information file. DOI: 10.1107/S2056989018006126/lh5872Isup3.cml

CCDC reference: 1838774

Additional supporting information:  crystallographic information; 3D view; checkCIF report

## Figures and Tables

**Figure 3:**
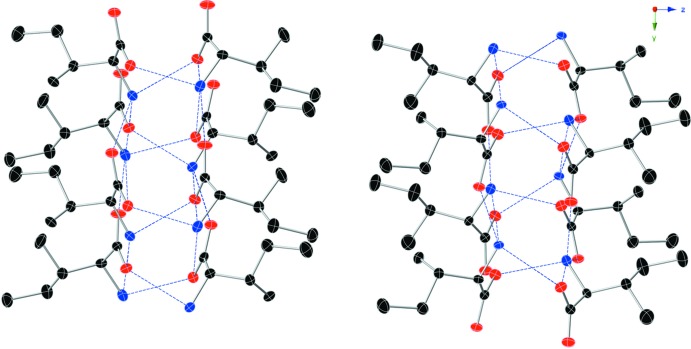
Part of the crystal structure viewed perpendicular to the *bc* plane showing adjacent *anti-parallel* layers formed by the hydrogen-bonded mol­ecule pairs and symmetry-related mol­ecules. The hydro­phobic side chains protrude away and are stacked together. Displacement ellipsoids are shown at the 50% probability level (C atoms black, O red, N blue). H atoms are omitted for clarity. Blue dashed lines denote hydrogen bonds.

**Table 1:** Hydrogen-bond geometry (Å, °)

*D*—H⋯*A*	*D*—H	H⋯*A*	*D*⋯*A*	*D*—H⋯*A*
N1—H1*A*⋯O2^i^	0.91	1.96	2.853 (5)	165
N3—H3*A*⋯O5^ii^	0.91	1.93	2.820 (5)	165
N3—H3*B*⋯O3^iii^	0.91	2.01	2.818 (5)	147
N3—H3*C*⋯O3^iv^	0.91	1.87	2.773 (4)	172
N4—H4*D*⋯O8^i^	0.91	1.97	2.843 (5)	162
N2—H2*A*⋯O5	0.91	2.19	3.055 (5)	159
N2—H2*A*⋯O6	0.91	2.20	2.953 (5)	139
N2—H2*C*⋯O6^v^	0.91	1.85	2.762 (5)	174
N2—H2*B*⋯O4^ii^	0.91	1.94	2.826 (5)	163

Symmetry codes: (i) 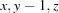; (ii) 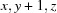; (iii) 
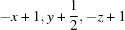
; (iv) 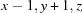; (v) 
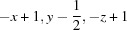
.

**Table 2:** Experimental details

Crystal data	
Chemical formula	C_6_H_13_NO_2_
*M*_r_	131.17
Crystal system, space group	Monoclinic, *P*2_1_
Temperature (K)	100
*a*, *b*, *c* (Å)	9.6757 (5), 5.2885 (3), 28.0136 (15)
β (°)	98.300 (3)
*V* (Å^3^)	1418.44 (13)
*Z*	8
Radiation type	Mo *K*α
μ (mm^−1^)	0.09
Crystal size (mm)	0.50 × 0.20 × 0.15

Data collection	
Diffractometer	Bruker APEXII KappaCCD
Absorption correction	Multi-scan (*SADABS*; Bruker, 2015 ▸)
*T*_min_, *T*_max_	0.956, 0.987
No. of measured, independent and observed [*I* > 2σ(*I*)] reflections	44938, 7935, 7188
*R*_int_	0.060
(sin θ/λ)_max_ (Å^−1^)	0.694

Refinement	
*R*[*F* ^2^ > 2σ(*F* ^2^)], *wR*(*F* ^2^), *S*	0.077, 0.211, 1.15
No. of reflections	7935
No. of parameters	338
No. of restraints	7
H-atom treatment	H-atom parameters constrained
Δρ_max_, Δρ_min_ (e Å^−3^)	0.58, −0.42
Absolute structure	Flack *x* determined using 2758 quotients [(*I* ^+^)−(*I* ^−^)]/[(*I* ^+^)+(*I* ^−^)] (Parsons *et al.*, 2013 ▸)
Absolute structure parameter	−0.2 (4)

Computer programs: *APEX2* and *SAINT* (Bruker, 2015 ▸), *SHELXT* (Sheldrick, 2015*a* ▸), *SHELXL2014* (Sheldrick, 2015*b* ▸), *PLATON* (Spek, 2009 ▸), *CrystalMaker* (*CrystalMaker*, 2013 ▸) and *Mercury* (Macrae *et al.*, 2008 ▸).

## supplementary crystallographic information

### Crystal data

**Table:**

C_6_H_13_NO_2_	*F*(000) = 576
*M**_r_* = 131.17	*D*_x_ = 1.228 Mg m^−^^3^
Monoclinic, *P*2_1_	Mo *K*α radiation, λ = 0.71073 Å
*a* = 9.6757 (5) Å	Cell parameters from 47498 reflections
*b* = 5.2885 (3) Å	θ = 0.7–30.6°
*c* = 28.0136 (15) Å	µ = 0.09 mm^−^^1^
β = 98.300 (3)°	*T* = 100 K
*V* = 1418.44 (13) Å^3^	Prism, colorless
*Z* = 8	0.50 × 0.20 × 0.15 mm

### Data collection

**Table:**

Bruker APEXII KappaCCD diffractometer	7188 reflections with *I* > 2σ(*I*)
φ and ω scans	*R*_int_ = 0.060
Absorption correction: multi-scan (SADABS; Bruker, 2015)	θ_max_ = 29.6°, θ_min_ = 2.8°
*T*_min_ = 0.956, *T*_max_ = 0.987	*h* = −13→13
44938 measured reflections	*k* = −7→7
7935 independent reflections	*l* = −38→38

### Refinement

**Table:**

Refinement on *F*^2^	H-atom parameters constrained
Least-squares matrix: full	*w* = 1/[σ^2^(*F*_o_^2^) + (0.0831*P*)^2^ + 2.4061*P*] where *P* = (*F*_o_^2^ + 2*F*_c_^2^)/3
*R*[*F*^2^ > 2σ(*F*^2^)] = 0.077	(Δ/σ)_max_ = 0.005
*wR*(*F*^2^) = 0.211	Δρ_max_ = 0.58 e Å^−^^3^
*S* = 1.15	Δρ_min_ = −0.42 e Å^−^^3^
7935 reflections	Extinction correction: SHELXL2014 (Sheldrick, 2015b), Fc^*^=kFc[1+0.001xFc^2^λ^3^/sin(2θ)]^-1/4^
338 parameters	Extinction coefficient: 0.043 (6)
7 restraints	Absolute structure: Flack *x* determined using 2758 quotients [(*I*^+^)-(*I*^-^)]/[(*I*^+^)+(*I*^-^)] (Parsons *et al.*, 2013)
Hydrogen site location: inferred from neighbouring sites	Absolute structure parameter: −0.2 (4)

### Special details

**Table:**

Geometry. All esds (except the esd in the dihedral angle between two l.s. planes) are estimated using the full covariance matrix. The cell esds are taken into account individually in the estimation of esds in distances, angles and torsion angles; correlations between esds in cell parameters are only used when they are defined by crystal symmetry. An approximate (isotropic) treatment of cell esds is used for estimating esds involving l.s. planes.

### Fractional atomic coordinates and isotropic or equivalent isotropic displacement parameters (Å^2^)

**Table:**

	*x*	*y*	*z*	*U*_iso_*/*U*_eq_	
O1	0.8884 (3)	0.5064 (6)	0.04413 (11)	0.0166 (6)	
O2	0.8218 (3)	0.8831 (6)	0.06814 (11)	0.0162 (6)	
C1	0.8005 (4)	0.6525 (8)	0.05824 (14)	0.0125 (7)	
C2	0.6598 (4)	0.5418 (8)	0.06684 (14)	0.0126 (7)	
H2	0.5843	0.6615	0.0531	0.015*	
N1	0.6375 (3)	0.2952 (7)	0.04012 (12)	0.0128 (6)	
H1A	0.7063	0.1847	0.0518	0.019*	
H1B	0.6396	0.3219	0.0081	0.019*	
H1C	0.5531	0.2297	0.0443	0.019*	
C3	0.6498 (4)	0.5036 (8)	0.12085 (15)	0.0155 (8)	
H3	0.5734	0.3791	0.1234	0.019*	
C4	0.6086 (5)	0.7558 (10)	0.14216 (16)	0.0223 (9)	
H4A	0.6805	0.8837	0.1379	0.027*	
H4B	0.5192	0.8135	0.1237	0.027*	
C5	0.5922 (6)	0.7431 (12)	0.19564 (18)	0.0311 (11)	
H5A	0.6832	0.7080	0.2148	0.047*	
H5B	0.5265	0.6080	0.2007	0.047*	
H5C	0.5566	0.9051	0.2057	0.047*	
C6	0.7850 (4)	0.3964 (9)	0.14852 (15)	0.0194 (8)	
H6A	0.7685	0.3426	0.1807	0.029*	
H6B	0.8576	0.5269	0.1516	0.029*	
H6C	0.8153	0.2510	0.1310	0.029*	
O3	0.8415 (3)	0.0087 (6)	0.45421 (11)	0.0164 (6)	
O4	0.7533 (3)	−0.3784 (6)	0.44210 (13)	0.0219 (7)	
C7	0.7422 (4)	−0.1469 (8)	0.44123 (14)	0.0142 (7)	
C8	0.6019 (4)	−0.0244 (8)	0.42024 (15)	0.0146 (8)	
H8	0.5250	−0.1492	0.4218	0.018*	
N2	0.5777 (4)	0.2014 (8)	0.44997 (13)	0.0172 (7)	
H2A	0.4852	0.2410	0.4452	0.026*	
H2B	0.6278	0.3345	0.4411	0.026*	
H2C	0.6053	0.1661	0.4817	0.026*	
C9	0.6022 (4)	0.0536 (9)	0.36746 (15)	0.0178 (8)	
H9	0.6802	0.1769	0.3666	0.021*	
C10	0.4661 (5)	0.1855 (10)	0.34710 (17)	0.0229 (9)	
H10A	0.4579	0.3436	0.3654	0.027*	
H10B	0.3868	0.0751	0.3521	0.027*	
C11	0.4548 (6)	0.2498 (11)	0.29339 (17)	0.0286 (11)	
H11A	0.4369	0.0949	0.2743	0.043*	
H11B	0.5424	0.3263	0.2869	0.043*	
H11C	0.3779	0.3692	0.2846	0.043*	
C12	0.6304 (8)	−0.1739 (12)	0.3372 (2)	0.0400 (15)	
H12A	0.6393	−0.1183	0.3044	0.060*	
H12B	0.5527	−0.2938	0.3360	0.060*	
H12C	0.7172	−0.2562	0.3516	0.060*	
O5	0.2587 (3)	0.1881 (6)	0.43174 (12)	0.0184 (6)	
O6	0.3479 (3)	0.5641 (6)	0.45466 (11)	0.0165 (6)	
C13	0.2467 (4)	0.4193 (8)	0.44065 (14)	0.0144 (8)	
C14	0.0998 (4)	0.5344 (8)	0.43171 (15)	0.0138 (7)	
H14	0.0340	0.4158	0.4448	0.017*	
N3	0.1002 (3)	0.7776 (7)	0.45836 (12)	0.0145 (7)	
H3A	0.1555	0.8911	0.4457	0.022*	
H3B	0.1338	0.7515	0.4900	0.022*	
H3C	0.0116	0.8390	0.4557	0.022*	
C15	0.0477 (4)	0.5754 (9)	0.37737 (15)	0.0162 (8)	
H15	−0.0310	0.6995	0.3749	0.019*	
C16	−0.0116 (5)	0.3277 (10)	0.35459 (17)	0.0241 (10)	
H16A	−0.0829	0.2629	0.3736	0.029*	
H16B	0.0645	0.2014	0.3567	0.029*	
C17	−0.0782 (6)	0.3523 (12)	0.30162 (19)	0.0325 (12)	
H17A	−0.0061	0.3961	0.2818	0.049*	
H17B	−0.1495	0.4853	0.2987	0.049*	
H17C	−0.1216	0.1912	0.2905	0.049*	
C18	0.1600 (5)	0.6889 (9)	0.35062 (16)	0.0192 (8)	
H18A	0.1184	0.7351	0.3178	0.029*	
H18B	0.2343	0.5644	0.3492	0.029*	
H18C	0.1993	0.8401	0.3677	0.029*	
O7	0.3780 (3)	0.0868 (6)	0.04550 (11)	0.0161 (6)	
O8	0.3022 (3)	0.4780 (6)	0.05701 (12)	0.0201 (7)	
C19	0.2915 (4)	0.2440 (8)	0.05879 (14)	0.0141 (7)	
C20	0.1698 (4)	0.1267 (8)	0.08112 (14)	0.0134 (7)	
H20	0.0926	0.2535	0.0795	0.016*	
N4	0.1169 (4)	−0.1027 (7)	0.05243 (13)	0.0156 (7)	
H4C	0.0295	−0.1410	0.0585	0.023*	
H4D	0.1749	−0.2357	0.0610	0.023*	
H4E	0.1143	−0.0702	0.0204	0.023*	
C21	0.2165 (4)	0.0562 (9)	0.13404 (15)	0.0169 (8)	
H21	0.2944	−0.0688	0.1352	0.020*	
C22	0.0973 (5)	−0.0696 (12)	0.15609 (18)	0.0301 (12)	
H22A	0.0153	0.0445	0.1517	0.036*	
H22B	0.0702	−0.2275	0.1382	0.036*	
C23	0.1336 (6)	−0.1325 (13)	0.20962 (18)	0.0325 (12)	
H23A	0.1303	0.0220	0.2287	0.049*	
H23B	0.2278	−0.2048	0.2157	0.049*	
H23C	0.0662	−0.2552	0.2188	0.049*	
C24	0.2721 (7)	0.2875 (13)	0.1629 (2)	0.0404 (14)	
H24A	0.1940	0.3971	0.1681	0.061*	
H24B	0.3357	0.3806	0.1450	0.061*	
H24C	0.3225	0.2338	0.1941	0.061*	

### Atomic displacement parameters (Å^2^)

**Table:**

	*U*^11^	*U*^22^	*U*^33^	*U*^12^	*U*^13^	*U*^23^
O1	0.0092 (12)	0.0188 (16)	0.0221 (15)	−0.0007 (11)	0.0029 (10)	−0.0017 (12)
O2	0.0148 (13)	0.0118 (14)	0.0225 (15)	−0.0048 (11)	0.0043 (11)	−0.0015 (12)
C1	0.0086 (16)	0.0147 (18)	0.0139 (16)	−0.0025 (13)	0.0001 (12)	0.0019 (14)
C2	0.0090 (15)	0.0120 (18)	0.0170 (17)	−0.0032 (14)	0.0020 (13)	−0.0007 (14)
N1	0.0104 (14)	0.0107 (15)	0.0165 (15)	−0.0019 (11)	−0.0011 (12)	0.0008 (12)
C3	0.0121 (16)	0.018 (2)	0.0159 (17)	−0.0024 (14)	0.0017 (13)	−0.0004 (15)
C4	0.024 (2)	0.024 (2)	0.021 (2)	0.0029 (18)	0.0066 (16)	−0.0020 (17)
C5	0.035 (3)	0.036 (3)	0.025 (2)	0.001 (2)	0.012 (2)	−0.006 (2)
C6	0.0179 (19)	0.023 (2)	0.0168 (18)	0.0006 (16)	0.0000 (15)	0.0030 (16)
O3	0.0109 (12)	0.0159 (15)	0.0218 (14)	0.0014 (11)	−0.0001 (10)	−0.0033 (11)
O4	0.0164 (15)	0.0124 (15)	0.0365 (19)	0.0049 (11)	0.0030 (13)	−0.0001 (13)
C7	0.0112 (17)	0.0148 (19)	0.0160 (18)	0.0024 (14)	−0.0001 (13)	−0.0005 (14)
C8	0.0094 (16)	0.0138 (18)	0.0205 (19)	−0.0011 (13)	0.0019 (14)	−0.0010 (15)
N2	0.0128 (15)	0.0223 (19)	0.0172 (16)	0.0095 (14)	0.0045 (12)	0.0038 (14)
C9	0.0129 (17)	0.021 (2)	0.0187 (19)	0.0011 (15)	−0.0004 (14)	0.0004 (16)
C10	0.0144 (19)	0.033 (3)	0.021 (2)	0.0019 (18)	0.0013 (15)	0.0027 (18)
C11	0.031 (2)	0.034 (3)	0.019 (2)	0.009 (2)	−0.0027 (17)	0.0027 (19)
C12	0.060 (4)	0.036 (3)	0.021 (2)	0.017 (3)	−0.003 (2)	−0.011 (2)
O5	0.0156 (14)	0.0139 (15)	0.0257 (15)	0.0046 (11)	0.0028 (11)	−0.0002 (12)
O6	0.0122 (13)	0.0194 (16)	0.0177 (14)	0.0013 (11)	0.0015 (10)	−0.0007 (12)
C13	0.0107 (16)	0.018 (2)	0.0139 (17)	0.0063 (14)	0.0017 (13)	0.0034 (14)
C14	0.0080 (15)	0.0159 (19)	0.0176 (18)	0.0008 (14)	0.0016 (13)	0.0012 (15)
N3	0.0118 (15)	0.0158 (17)	0.0166 (16)	0.0020 (13)	0.0043 (12)	0.0028 (13)
C15	0.0119 (17)	0.020 (2)	0.0162 (18)	0.0027 (15)	−0.0004 (14)	−0.0009 (15)
C16	0.020 (2)	0.027 (2)	0.023 (2)	−0.0042 (18)	−0.0031 (16)	−0.0015 (18)
C17	0.028 (2)	0.040 (3)	0.027 (2)	−0.003 (2)	−0.0058 (19)	−0.007 (2)
C18	0.0201 (19)	0.017 (2)	0.0203 (19)	0.0000 (16)	0.0041 (15)	0.0056 (16)
O7	0.0101 (12)	0.0177 (15)	0.0214 (14)	−0.0005 (11)	0.0048 (10)	−0.0034 (11)
O8	0.0194 (15)	0.0144 (15)	0.0273 (16)	−0.0022 (12)	0.0060 (12)	0.0006 (12)
C19	0.0097 (16)	0.0160 (19)	0.0161 (17)	−0.0004 (14)	0.0007 (13)	0.0008 (14)
C20	0.0122 (17)	0.0145 (18)	0.0135 (17)	0.0011 (13)	0.0023 (13)	0.0024 (14)
N4	0.0114 (15)	0.0192 (18)	0.0155 (15)	−0.0044 (13)	0.0001 (12)	0.0018 (13)
C21	0.0134 (17)	0.020 (2)	0.0171 (18)	−0.0005 (15)	0.0022 (14)	0.0010 (15)
C22	0.016 (2)	0.051 (3)	0.024 (2)	0.000 (2)	0.0035 (17)	0.010 (2)
C23	0.034 (3)	0.045 (3)	0.020 (2)	−0.005 (2)	0.0069 (19)	0.006 (2)
C24	0.051 (2)	0.037 (2)	0.032 (2)	−0.010 (2)	0.0032 (19)	−0.0004 (18)

### Geometric parameters (Å, º)

**Table:**

O1—C1	1.255 (5)	O5—C13	1.256 (5)
O2—C1	1.261 (5)	O6—C13	1.260 (5)
C1—C2	1.532 (5)	C13—C14	1.534 (5)
C2—N1	1.504 (5)	C14—N3	1.487 (5)
C2—C3	1.543 (6)	C14—C15	1.548 (6)
C2—H2	1.0000	C14—H14	1.0000
N1—H1A	0.9100	N3—H3A	0.9100
N1—H1B	0.9100	N3—H3B	0.9100
N1—H1C	0.9100	N3—H3C	0.9100
C3—C6	1.530 (6)	C15—C18	1.529 (6)
C3—C4	1.537 (6)	C15—C16	1.532 (6)
C3—H3	1.0000	C15—H15	1.0000
C4—C5	1.530 (7)	C16—C17	1.535 (7)
C4—H4A	0.9900	C16—H16A	0.9900
C4—H4B	0.9900	C16—H16B	0.9900
C5—H5A	0.9800	C17—H17A	0.9800
C5—H5B	0.9800	C17—H17B	0.9800
C5—H5C	0.9800	C17—H17C	0.9800
C6—H6A	0.9800	C18—H18A	0.9800
C6—H6B	0.9800	C18—H18B	0.9800
C6—H6C	0.9800	C18—H18C	0.9800
O3—C7	1.277 (5)	O7—C19	1.273 (5)
O4—C7	1.229 (5)	O8—C19	1.243 (5)
C7—C8	1.542 (5)	C19—C20	1.541 (5)
C8—N2	1.493 (6)	C20—N4	1.504 (5)
C8—C9	1.536 (6)	C20—C21	1.532 (6)
C8—H8	1.0000	C20—H20	1.0000
N2—H2A	0.9100	N4—H4C	0.9100
N2—H2B	0.9100	N4—H4D	0.9100
N2—H2C	0.9100	N4—H4E	0.9100
C9—C12	1.519 (7)	C21—C24	1.521 (8)
C9—C10	1.526 (6)	C21—C22	1.536 (6)
C9—H9	1.0000	C21—H21	1.0000
C10—C11	1.531 (6)	C22—C23	1.526 (7)
C10—H10A	0.9900	C22—H22A	0.9900
C10—H10B	0.9900	C22—H22B	0.9900
C11—H11A	0.9800	C23—H23A	0.9800
C11—H11B	0.9800	C23—H23B	0.9800
C11—H11C	0.9800	C23—H23C	0.9800
C12—H12A	0.9800	C24—H24A	0.9800
C12—H12B	0.9800	C24—H24B	0.9800
C12—H12C	0.9800	C24—H24C	0.9800
			
O1—C1—O2	124.6 (4)	O5—C13—O6	124.2 (4)
O1—C1—C2	118.2 (4)	O5—C13—C14	117.7 (4)
O2—C1—C2	117.1 (4)	O6—C13—C14	118.0 (4)
N1—C2—C1	108.7 (3)	N3—C14—C13	109.0 (3)
N1—C2—C3	110.5 (3)	N3—C14—C15	110.5 (3)
C1—C2—C3	112.8 (3)	C13—C14—C15	112.2 (3)
N1—C2—H2	108.3	N3—C14—H14	108.3
C1—C2—H2	108.3	C13—C14—H14	108.3
C3—C2—H2	108.3	C15—C14—H14	108.3
C2—N1—H1A	109.5	C14—N3—H3A	109.5
C2—N1—H1B	109.5	C14—N3—H3B	109.5
H1A—N1—H1B	109.5	H3A—N3—H3B	109.5
C2—N1—H1C	109.5	C14—N3—H3C	109.5
H1A—N1—H1C	109.5	H3A—N3—H3C	109.5
H1B—N1—H1C	109.5	H3B—N3—H3C	109.5
C6—C3—C4	112.1 (4)	C18—C15—C16	112.4 (4)
C6—C3—C2	112.0 (3)	C18—C15—C14	112.6 (3)
C4—C3—C2	108.9 (3)	C16—C15—C14	109.8 (4)
C6—C3—H3	107.9	C18—C15—H15	107.2
C4—C3—H3	107.9	C16—C15—H15	107.2
C2—C3—H3	107.9	C14—C15—H15	107.2
C5—C4—C3	114.3 (4)	C15—C16—C17	114.2 (4)
C5—C4—H4A	108.7	C15—C16—H16A	108.7
C3—C4—H4A	108.7	C17—C16—H16A	108.7
C5—C4—H4B	108.7	C15—C16—H16B	108.7
C3—C4—H4B	108.7	C17—C16—H16B	108.7
H4A—C4—H4B	107.6	H16A—C16—H16B	107.6
C4—C5—H5A	109.5	C16—C17—H17A	109.5
C4—C5—H5B	109.5	C16—C17—H17B	109.5
H5A—C5—H5B	109.5	H17A—C17—H17B	109.5
C4—C5—H5C	109.5	C16—C17—H17C	109.5
H5A—C5—H5C	109.5	H17A—C17—H17C	109.5
H5B—C5—H5C	109.5	H17B—C17—H17C	109.5
C3—C6—H6A	109.5	C15—C18—H18A	109.5
C3—C6—H6B	109.5	C15—C18—H18B	109.5
H6A—C6—H6B	109.5	H18A—C18—H18B	109.5
C3—C6—H6C	109.5	C15—C18—H18C	109.5
H6A—C6—H6C	109.5	H18A—C18—H18C	109.5
H6B—C6—H6C	109.5	H18B—C18—H18C	109.5
O4—C7—O3	125.2 (4)	O8—C19—O7	125.2 (4)
O4—C7—C8	119.7 (4)	O8—C19—C20	119.3 (4)
O3—C7—C8	115.0 (4)	O7—C19—C20	115.4 (4)
N2—C8—C9	110.2 (3)	N4—C20—C21	110.5 (3)
N2—C8—C7	108.9 (3)	N4—C20—C19	109.2 (3)
C9—C8—C7	110.9 (3)	C21—C20—C19	110.8 (3)
N2—C8—H8	109.0	N4—C20—H20	108.8
C9—C8—H8	109.0	C21—C20—H20	108.8
C7—C8—H8	109.0	C19—C20—H20	108.8
C8—N2—H2A	109.5	C20—N4—H4C	109.5
C8—N2—H2B	109.5	C20—N4—H4D	109.5
H2A—N2—H2B	109.5	H4C—N4—H4D	109.5
C8—N2—H2C	109.5	C20—N4—H4E	109.5
H2A—N2—H2C	109.5	H4C—N4—H4E	109.5
H2B—N2—H2C	109.5	H4D—N4—H4E	109.5
C12—C9—C10	111.6 (4)	C24—C21—C20	110.5 (4)
C12—C9—C8	110.5 (4)	C24—C21—C22	111.3 (4)
C10—C9—C8	111.2 (3)	C20—C21—C22	111.2 (3)
C12—C9—H9	107.8	C24—C21—H21	107.9
C10—C9—H9	107.8	C20—C21—H21	107.9
C8—C9—H9	107.8	C22—C21—H21	107.9
C9—C10—C11	113.8 (4)	C23—C22—C21	114.2 (4)
C9—C10—H10A	108.8	C23—C22—H22A	108.7
C11—C10—H10A	108.8	C21—C22—H22A	108.7
C9—C10—H10B	108.8	C23—C22—H22B	108.7
C11—C10—H10B	108.8	C21—C22—H22B	108.7
H10A—C10—H10B	107.7	H22A—C22—H22B	107.6
C10—C11—H11A	109.5	C22—C23—H23A	109.5
C10—C11—H11B	109.5	C22—C23—H23B	109.5
H11A—C11—H11B	109.5	H23A—C23—H23B	109.5
C10—C11—H11C	109.5	C22—C23—H23C	109.5
H11A—C11—H11C	109.5	H23A—C23—H23C	109.5
H11B—C11—H11C	109.5	H23B—C23—H23C	109.5
C9—C12—H12A	109.5	C21—C24—H24A	109.5
C9—C12—H12B	109.5	C21—C24—H24B	109.5
H12A—C12—H12B	109.5	H24A—C24—H24B	109.5
C9—C12—H12C	109.5	C21—C24—H24C	109.5
H12A—C12—H12C	109.5	H24A—C24—H24C	109.5
H12B—C12—H12C	109.5	H24B—C24—H24C	109.5
			
O1—C1—C2—N1	−19.5 (5)	O5—C13—C14—N3	162.0 (4)
O2—C1—C2—N1	163.7 (3)	O6—C13—C14—N3	−20.6 (5)
O1—C1—C2—C3	103.3 (4)	O5—C13—C14—C15	−75.3 (5)
O2—C1—C2—C3	−73.5 (5)	O6—C13—C14—C15	102.1 (4)
N1—C2—C3—C6	80.1 (4)	N3—C14—C15—C18	78.1 (4)
C1—C2—C3—C6	−41.7 (5)	C13—C14—C15—C18	−43.8 (5)
N1—C2—C3—C4	−155.4 (3)	N3—C14—C15—C16	−155.8 (3)
C1—C2—C3—C4	82.8 (4)	C13—C14—C15—C16	82.3 (4)
C6—C3—C4—C5	−56.6 (5)	C18—C15—C16—C17	−58.9 (5)
C2—C3—C4—C5	178.9 (4)	C14—C15—C16—C17	174.9 (4)
O4—C7—C8—N2	141.7 (4)	O8—C19—C20—N4	141.5 (4)
O3—C7—C8—N2	−41.9 (5)	O7—C19—C20—N4	−41.7 (5)
O4—C7—C8—C9	−96.9 (5)	O8—C19—C20—C21	−96.5 (5)
O3—C7—C8—C9	79.4 (5)	O7—C19—C20—C21	80.3 (4)
N2—C8—C9—C12	178.6 (4)	N4—C20—C21—C24	179.1 (4)
C7—C8—C9—C12	58.0 (5)	C19—C20—C21—C24	57.9 (5)
N2—C8—C9—C10	−56.9 (5)	N4—C20—C21—C22	−56.8 (5)
C7—C8—C9—C10	−177.5 (4)	C19—C20—C21—C22	−178.0 (4)
C12—C9—C10—C11	−51.6 (6)	C24—C21—C22—C23	−53.2 (6)
C8—C9—C10—C11	−175.4 (4)	C20—C21—C22—C23	−176.9 (5)

### Hydrogen-bond geometry (Å, º)

**Table:**

*D*—H···*A*	*D*—H	H···*A*	*D*···*A*	*D*—H···*A*
N1—H1*A*···O2^i^	0.91	1.96	2.853 (5)	165
N3—H3*A*···O5^ii^	0.91	1.93	2.820 (5)	165
N3—H3*B*···O3^iii^	0.91	2.01	2.818 (5)	147
N3—H3*C*···O3^iv^	0.91	1.87	2.773 (4)	172
N4—H4*D*···O8^i^	0.91	1.97	2.843 (5)	162
N2—H2*A*···O5	0.91	2.19	3.055 (5)	159
N2—H2*A*···O6	0.91	2.20	2.953 (5)	139
N2—H2*C*···O6^v^	0.91	1.85	2.762 (5)	174
N2—H2*B*···O4^ii^	0.91	1.94	2.826 (5)	163

Symmetry codes: (i) *x*, *y*−1, *z*; (ii) *x*, *y*+1, *z*; (iii) −*x*+1, *y*+1/2, −*z*+1; (iv) *x*−1, *y*+1, *z*; (v) −*x*+1, *y*−1/2, −*z*+1.
